# Transcriptomic profiles reveal the genome-wide responses of the harmful dinoflagellate *Cochlodinium polykrikoides* when exposed to the algicide copper sulfate

**DOI:** 10.1186/s12864-015-2341-3

**Published:** 2016-01-05

**Authors:** Ruoyu Guo, Hui Wang, Young Sang Suh, Jang-Seu Ki

**Affiliations:** Department of Life Science, College of Natural Sciences, Sangmyung University, Seoul, 110-743 Korea; Fishery and Ocean Information Division, National Fisheries Research & Development Institute, Busan, 619-705 Korea

**Keywords:** *Cochlodinium polykrikoides*, Algicide CuSO_4_, Trancriptomic response, Differentially expressed genes

## Abstract

**Background:**

Harmful algal blooms (HABs) caused by the dinoflagellate *Cochlodinium polykrikoides* lead to severe environmental impacts in oceans worldwide followed by huge economic losses. Algicide agent copper sulfate (CuSO_4_) is regard as an economical and effective agent for HABs mitigation; its biochemical and physiological effects were revealed in *C. polykrikoides*. However, molecular mechanisms of CuSO_4_ effect on the *C. polykrikoides*, even other HAB species, have not been investigated. The present study investigated the transcriptional response of *C. polykrikoides* against CuSO_4_ treatments, with the aim of providing certain molecular mechanism of CuSO_4_ effect on the *C. polykrikoides* blooms.

**Results:**

RNA-seq generated 173 million reads, which were further assembled to 191,212 contigs. 43.3 %, 33.9 %, and 15.6 % of contigs were annotated with NCBI NR, GO, and KEGG database, respectively. Transcriptomic analysis revealed 20.6 % differential expressed contigs, which grouped into 8 clusters according to *K*-means clustering analysis, responding to CuSO_4_; 848 contigs were up-regulated and 746 contigs were down-regulated more than 2-fold changes from 12 h to 48 h exposure. KEGG pathway analysis of eukaryotic homologous genes revealed the differentially expressed genes (DEGs) were involved in diverse pathway; amongst, the genes involved in the translation, spliceosome, and/or signal transduction genes were highly regulated. Most of photosystem related genes were down-regulated and most of mitochondria related genes were up-regulated. In addition, the genes involved in the copper ion binding or transporting and antioxidant systems were identified. Measurement of chlorophyll fluorescence showed that photosynthesis was significantly inhibited by CuSO_4_ exposure.

**Conclusions:**

This study reported the first transcriptome of the *C. polykrikoides*. The widely differential expressed photosystem genes suggested photosynthetic machinery were severely affected, and may further contribute to the cell death. Furthermore, gene translation and transcription processes may be disrupted, inhibiting cell growth and proliferation, and possibly accelerating cell death. However, antioxidant systems resistant to CuSO_4_ caused stress; mitochondrion may compensate for photosynthesis efficiency decreasing caused energy deficiency. In addition, various signal transduction pathways may be involved in the CuSO_4_ induced regulation network in the *C. polykrikoides*. These data provide the potential transcriptomic mechanism to explain the algicide CuSO_4_ effect on the harmful dinoflagellate *C. polykrikoides*.

**Electronic supplementary material:**

The online version of this article (doi:10.1186/s12864-015-2341-3) contains supplementary material, which is available to authorized users.

## Background

Dinoflagellates are a large group of freshwater and marine microalgae; about half of them are photosynthetic, and thus they play a crucial role in aquatic ecosystems. To date, approximately 4500 dinoflagellate species have been described, including more than 2500 extinct species from the fossil record and approximately 2000 living species [1]. Some species are responsible for harmful algal blooms (HABs, sometimes referred to as red-tide), which can severely affect aquaculture and marine environments worldwide. Hence, much effort has been directed at trying to solve problems associated with HABs, including the causes of occurrence, identification of the organisms responsible, bloom dynamics, toxin production and associated genetics, environmental monitoring, management [2–4]. Some dinoflagellates (e.g., *Alexandrium tarmarense*, *Gymnodinium breve*, and *Prorocentrum minimum*) can produce toxins that affect fish, shellfish, mammals, seabirds, and other consumers by persisting in the food chain. In addition, large numbers of dinoflagellate cells clog gills and/or deplete oxygen levels in the water column, sometimes leading to massive morality of marine animals [5, 6]. To reduce these adverse effects, people have employed biological, chemical and physical approaches to control, prevent, and/or mitigate HABs [7, 8]. Amongst these, algicides that affect algae growth such as yellow loess, copper sulfate (CuSO_4_), hydrogen peroxide (H_2_O_2_), and oxidizing chlorine (Cl_2_) are regarded as effective ways to manage algal blooms, and can be applied in doses considered safe for environmental health [7, 9, 10]. To date, studies on the effects of these algicides on HAB species have mainly focused on the biocidal efficiency, by measuring cell growth, pigment content, and photosynthetic efficiency. Recently cellular and biochemical responses of functional genes, such as those involved in photosynthesis, to algicides have been assessed [9, 11, 12].

As unicellular microeukaryotes, dinoflagellates have distinct genomic characters (e.g., permanently condensed and liquid-crystalline chromosomes, very large nuclear genome sizes, low amounts of histones, ~70 % replacement of thymine with 5-hydroxymethyluracil, etc.). These properties make dinoflagellates an interesting model for genomic research [13]. In addition, some dinoflagellates lack typical eukaryotic transcriptional elements (e.g., TATA boxes) in the upstream regions of coding genes [14]. Hence, they may have specific regulatory mechanisms of gene expression (e.g., spliced leader *trans*-splicing, post-transcriptional regulation, etc.). Furthermore, dinoflagellate spliced-leader (dinoSL) *trans*-splicing is known to be a common transcription mechanism in nuclear genomes [15]. However, recent studies have demonstrated that *Symbiodinium minutum* differs from other dinoflagellates in that not all its nuclear genes are dinoSL *trans*-splicing [15, 16]. Moreover, Brunelle and Van Dolah [17] found that cell cycle-related genes (e.g., those responsible for producing cell nuclear antigens, ribonucleotide reductase, and replication factor C) were not altered at transcriptional level but at the protein level during the cell cycle in *Karenia brevis*. Hence, they proposed that expression of these genes was regulated post-transcription in this dinoflagellate. To the best of our knowledge, the transcriptional responses of dinoflagellate genes vary widely in different environmental conditions [18–20]. These findings show that dinoflagellate genes regulation may be affected by environmental changes.

From a molecular perspective, the large genomes of HAB-forming dinoflagellates implies that they should have specific duplication mechanisms (e.g., a permanently present nuclear membrane, even during mitosis) to allow rapid proliferation, especially during bloom initiation. Therefore, dinoflagellate genome and transcriptional studies may help in understanding these molecular mechanisms in HAB-forming species. Recently developed molecular methods (e.g., next generation sequencing and microarrays) have been applied to investigate the genome and transcriptome characteristics of HAB-forming species [16, 18, 20–23]. In addition, the roles of gene expression and regulation in mediating the effects of nutrient availability on HAB-forming dinoflagellate growth have been studied [20, 24]. Despite these advances, few studies have examined the molecular mechanisms leading to HAB termination, particularly the genome-wide gene responses to algicides

The marine dinoflagellate *Cochlodinium polykrikoides* is widely distributed in tropical and temperate zones throughout the world (see review by Kudela and Gobler [25]). The species causes fish mortality by producing massive amounts of mucous and depleting dissolved oxygen [26]. The HABs formed by *C. polykrikoides* lead to serious economic losses and environmental impacts. They are highly toxic to organisms that feed upon them, especially fish [26, 27]. Recently, *C. polykrikoides* has spread to many oceanic regions, including Europe, India, the Middle East, and North America [25]. Many studies on the species have been carried out in the last three decades, including environmental surveys, studies aimed at mitigation of harmful effects, those documenting global expansion, etc. However, studies on the effects of algicides at the cellular and genome level in *C. polykrikoides*, or other harmful dinoflagellates, are lacking. Such studies are necessary to understand the molecular mechanisms underlying bloom initiation, expansion, and termination.

In recent studies, we examined the physiological responses of *C. polykrikoides* to a common biocide (hereafter referred to as algicide), CuSO_4_, and found significant decreases of cell number and the pigment content as well as chlorophyll autofluorescence intensity [10]. These results indicate that the algicide had a considerable effect on *C. polykrikoides* at the cellular level, even greater than that of other chemicals such as yellow loess (unpublished data). In this study, we tested the effects of the algicide copper sulfate on the transcriptional responses of *C. polykrikoides* to understand its effects at the molecular level. First, we obtained large-scale cDNA sequences for *C. polykrikoides*, investigated the transcripts of cells exposed to copper sulfate, and then characterized these with bioinformatics tools, including the NCBI non-redundant protein (NR) database, the gene ontology (GO) database, and the Kyoto Encyclopedia of Genes and Genomes (KEGG) database. In addition, we investigated the transcriptome response to determine how copper sulfate affects *C. polykrikoides* at the genomic level and what kinds of gene regulation mechanisms are involved its defensive response.

## Methods

### Culture and algicide treatment

A strain (CP-01) of *C. polykrikoides* was obtained from the National Fisheries Research and Development Institute (NFRDI) of Korea, and cultured in f/2 medium at 20 °C under a 12:12-h light–dark cycle with a photon flux density of about 65 μmol photons m^−2^ s^−1^.

At the exponential growth phase, cells of *C. polykrikoides* were exposed to the algicide copper sulfate (CuSO_4_, Cat. No. C1297, Sigma, MO) with final concentration of 1 mg L^−1^. The CuSO_4_ concentration used in this study was selected according to the median effective concentration value (EC_50_ value) tested by [28], which was 10 times lower than reported EC_50_ value. The exposed cultures were harvested at 12 h, 24 h, and 48 h, and the untreated cells were used as control.

### RNA extraction and cDNA library construction

*C. polykrikoides* cultures were harvested using centrifugation at 1000 *g* for 6 min, immediately frozen in liquid nitrogen, and stored at −80 °C until RNA extraction. Preserved cells were physically broken by freeze-thawing in liquid nitrogen, and further homogenized with a mini-beadbeater (BioSpec Products Inc., Bartlesville, OK) with zirconium beads (0.1 mm diameter). Total RNA was isolated using TRIzol (Invitrogen, Carlsbad, CA), and purified using Mini Spin Columns from RNeasy Mini Kits (Qiagen, Valencia, CA). Total RNA integrity and quality were checked using an Agilent 2100 Bioanalyzer (Aglient, Santa Clara, CA). The cDNA library for subsequent cluster generation was prepared using the reagents provided in the Illumina ® TruSeq™ RNA Sample Preparation Kit (RS-122-2001, Illumina Inc., San Diego, CA). Sequencing was finished by a commercial service (Macrogen Inc., Seoul, Korea) using the Illumina HiSeq 2500 (Illumina Inc., San Diego, CA).

### Transcriptome assembly and functional annotation

The quality of raw reads was checked with FastQC_v0.10.0 (http://www.bioinformatics.bbsrc.ac.uk/projects/fastqc/). The raw data were cleaned and trimmed by removing adaptor and low quality reads, and then reads were assembled using Trinity software [29]. The contigs were annotated by BLASTX alignment, with *E*-value < 0.001, against the NCBI non-redundant protein (NR) database. For pathway enrichment analysis, the contigs were assigned to the Kyoto Encyclopedia of Genes and Genomes (KEGG) database [30] using the single-directional best hit (SBH) method contained in the online tool, KEGG Automatic Annotation Server. Functional annotation of contigs by gene ontology (GO) was carried out with Blast2go software [31]. The raw read transcriptome sequences were submitted to the NCBI Sequence Read Archive database (accession number SRR1548539).

### Analysis of differentially expressed genes

The gene expression levels of the contigs were calculated as fragments per kilobase of transcript per million mapped reads (FPKM). The degree of differential gene expression in CuSO_4_ treated samples was calculated by comparison to the control (untreated exponential growth phase samples). The Log_2_ ratio ≥ 1 (fold change ≥ 2) was used as the threshold to define significantly differentially expressed genes (DEGs). All the DEGs were analyzed by clustering algorithm analysis using *K*-mean clustering. The identified eukaryote DEGs were mapped to the GO and KEGG databases. In the KEGG analysis, KEGG database assignment showed that some contigs coded for the same proteins, and the KEGG pathway analysis accounted for this when calculating the number of coded proteins. Contigs that coded for the same protein were considered as single genes, and were counted as one. In addition, the DEGs were further manually characterized with GO and NR database annotation by reviewing previous studies. The assembled sequences and raw FPKM values were registered in the GEO database with an accession number GSE75463.

### Photosynthesis and oxidative stress measurements

Chlorophyll fluorescence was measured using a Handy PEA (Hansatech Instruments Ltd, Norfolk, UK). The parameters *F*o, *F*v, and *F*m were measured at 0 h, 12 h, 24 h, and 48 h after CuSO_4_ exposure. The ratios *F*v/*F*m and *F*v/*F*o were calculated; *F*v/*F*m is an indicator of the photosynthetic efficiency, and *F*v/*F*o is a measure of the activity of the water-splitting complex on the donor side of photosystem II, as well as the size and number of active photosynthetic reaction centers [32, 33]. In addition, the maximum yield of primary photochemistry (ΨEo = TRo/ABS), and efficiency with which a trapped exciton can move an electron into the electron transport chain further than Q_A_- (Ψo = Eto/Tro) were also calculated using a Handy PEA.

To detect reactive oxygen species (ROS), cells were stained with DHR123 (D1054; Sigma) for 1 h. The DHR123 stock solutions were directly added into cell cultures at a final concentration of 5 μM/L. After incubation, cellular ROS content was measured with an LS-55 fluorescence spectrometer (Perkin-Elmer, Waltham, MA). Lipid peroxidation was measured according to the method described in [34]. One-way analysis of variance (ANOVA) with post hoc Dunnett’s multiple comparison test using Graphpad InStat (Graphpad Software, Inc., USA) was used for comparisons between control and treated cultures. Data are represented as mean ± SD, and *P* < 0.05 was considered statistically significant.

### Quantitative real-time PCR for gene validation

Some DEGs of interest were selected and validated using quantitative real-time PCR (qRT-PCR). The primers used in the qRT-PCR are listed in Additional file 1. All qRT-PCR reactions were performed with TOPreal™ qPCR 2X PreMIX (TOP, enzynomics, Korea) in a CFX96 Real-Time PCR Detection System (Bio-Rad; Hercules, CA). The qRT-PCR conditions were as follows: 4 min at 50 °C; 10 min at 95 °C, followed by 40 cycles of 10 s at 95 °C, 15 s at 60 °C, and 15 s at 72 °C. All reactions were performed in triplicate, and the mean value was calculated. The specificity of the amplification was verified through the analysis of a melting curve generated by gradually heating the sample from 65 °C to 95 °C. The α-tubulin (*TUA*) gene, which has the most stable expression pattern known in the dinoflagellate *Prorocentrum minimum* [35], was used as an internal control. *C*_T_ values of qRT-PCR were obtained using CFX96 Real-Time controlling software (Bio-Rad; Hercules, CA). The fold-change relative to control was calculated according to the method of Pfaffl [36]. The Spearman correlation coefficient of the gene expression results from Hiseq2500 sequencing and qRT-PCR were calculated with Origin 8 software (OriginLab Corporation, MA).

## Results

### Transcriptome and functional gene annotations

To profile the transcriptome of *C. polykrikoides*, we constructed cDNA libraries, including CuSO_4_ exposed samples (at 12 h, 24 h, and 48 h), and unexposed control sample. RNA sequencing generated 173 million reads from libraries, containing 26.1 Gb nucleotides. The raw reads were assembled with 90 % similarity and 191,212 contigs with a mean length of 922 bp (Table 1). Contig length ranged from 201 to 36,127 bp (Table 1; Additional file 2). Of these contigs, 102,744 (53.7 %) were 201–600 bp in length, 38,593 (20.2 %) were 601-bp, 23,889 (12.5 %) were 1201–1800 bp, and 25,986 (19.0 %) were longer than 1800 bp.

**Table 1:** Summary of the *Cochlodinium polykrikoides* transcriptome

Category	Number	N50 (bp)	Total nucleotides (bp)	Maximum length (bp)	Minimum length (bp)
Read	172,977,960	-	26,119,671,960	-	-
Contigs	191,212	1550	176,356,262	36,127	201

In addition, individual contigs were assigned to three different protein databases: NCBI NR, Gene Ontology (GO), and Kyoto Encyclopedia of Genes and Genomes (KEGG) (Table 2). All contigs were aligned on the NCBI NR protein database by BLASTX with an *E*-value cutoff of 10^−4^. Of the 191,212 contigs, a total of 82,749 (43.28 %) were annotated in the NCBI NR database. Of these annotated contigs, 82.5 % were assigned to eukaryotes, 16.4 % were assigned to bacteria, and 1.1 % were classified as ‘other’. The superphylum Alveolata accounted for 16.8 % of annotated contigs, including 3.6 % Apicomplexa, 2.1 % Ciliophora, 3.6 % Dinophyta, and 7.6 % Perkinsus. In addition, the same sequences were analyzed to the GO database, and 64,931 contigs (33.96 %) were annotated. Overall, the annotations for these contigs were similar to those obtained from NCBI NR.

**Table 2:** Summary of annotation of contigs in each database

Public protein database	Number of contigs hits	Percentage (%)
NCBI NR	82,749	43.28 %
GO	64,931	33.96 %
KEGG	29,983	15.60 %

**Fig. 1:**
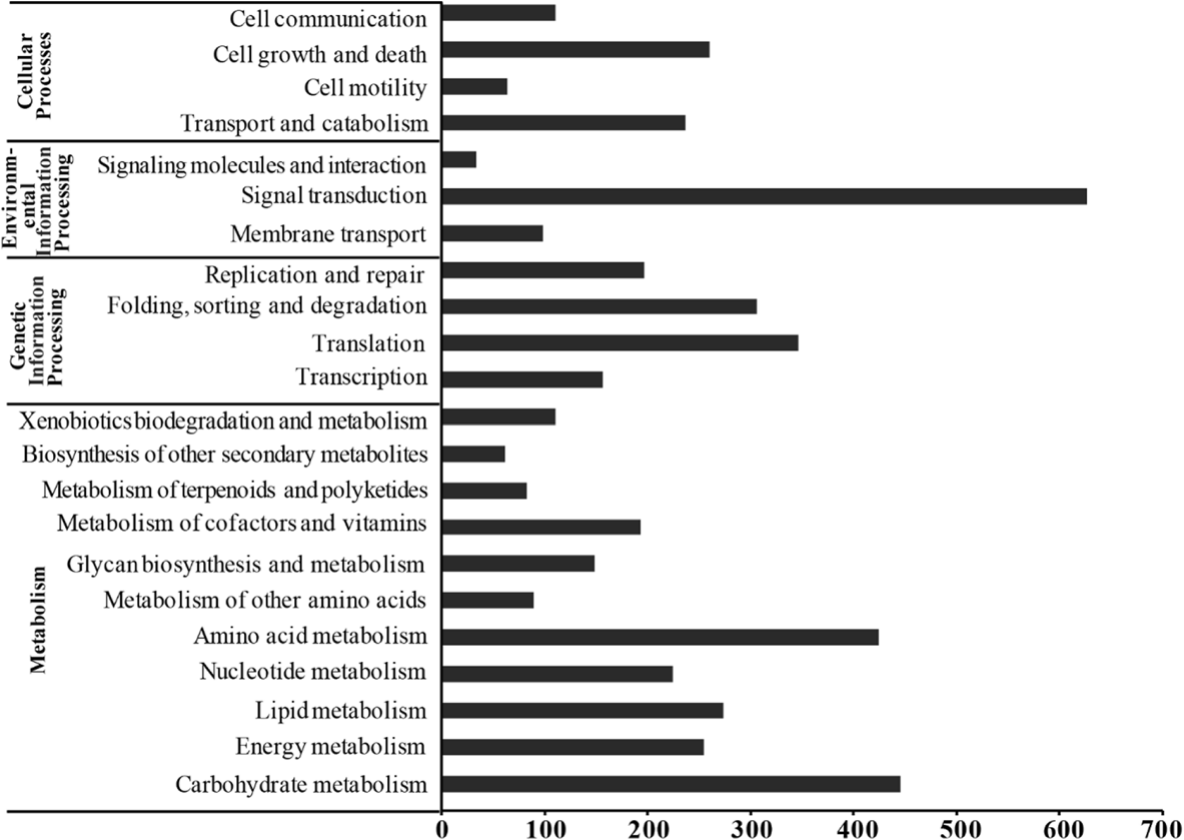
Pathway assignment based on KEGG pathway analysis. The gene numbers were obtained from KEGG pathway online analysis

In the KEGG analysis, a total of 29,983 contigs (15.6 %) were assigned to 217 KEGG pathways, excluding organismal systems and human diseases. Of these, 2297 genes were assigned to metabolic processing; 1007 to genetic information processing; 717 to environmental information processing; 666 to cellular processes (Fig. 1). The highest number of genes (586) was assigned to signal transduction, followed by carbohydrate metabolism (447 genes) and amino acid metabolism (418 genes). Furthermore, within the third level KEGG pathway (Additional file 3), the highest number of genes (126) was assigned to ribosome, followed by purine metabolism (125 genes), spliceosome (104 genes), and pyrimidine metabolism (99 genes) (Fig. 1).

### Clustering of DEGs following algicide exposure

Differential expression of genes in algicide-exposed cells was evaluated using the abundance of transcripts, quantified as FPKM. Contigs with FPKM lower than zero of were excluded from DEG analyses. Based on this cut-off threshold, a total of 100,370 contigs were excluded, and 90,842 contigs were retained in subsequent analyses. Of these, 18,700 contigs (around 20.6 %) showed differentially expressed patterns, as determined by 2-fold changes in expression. Of contigs with 2-fold or greater changes in expression, 3816, 3430, and 5792 were up-regulated and 5304, 2530, 6052 were down-regulated at 12 h, 24 h, and 48 h, respectively (Additional file 4).

**Fig. 2:**
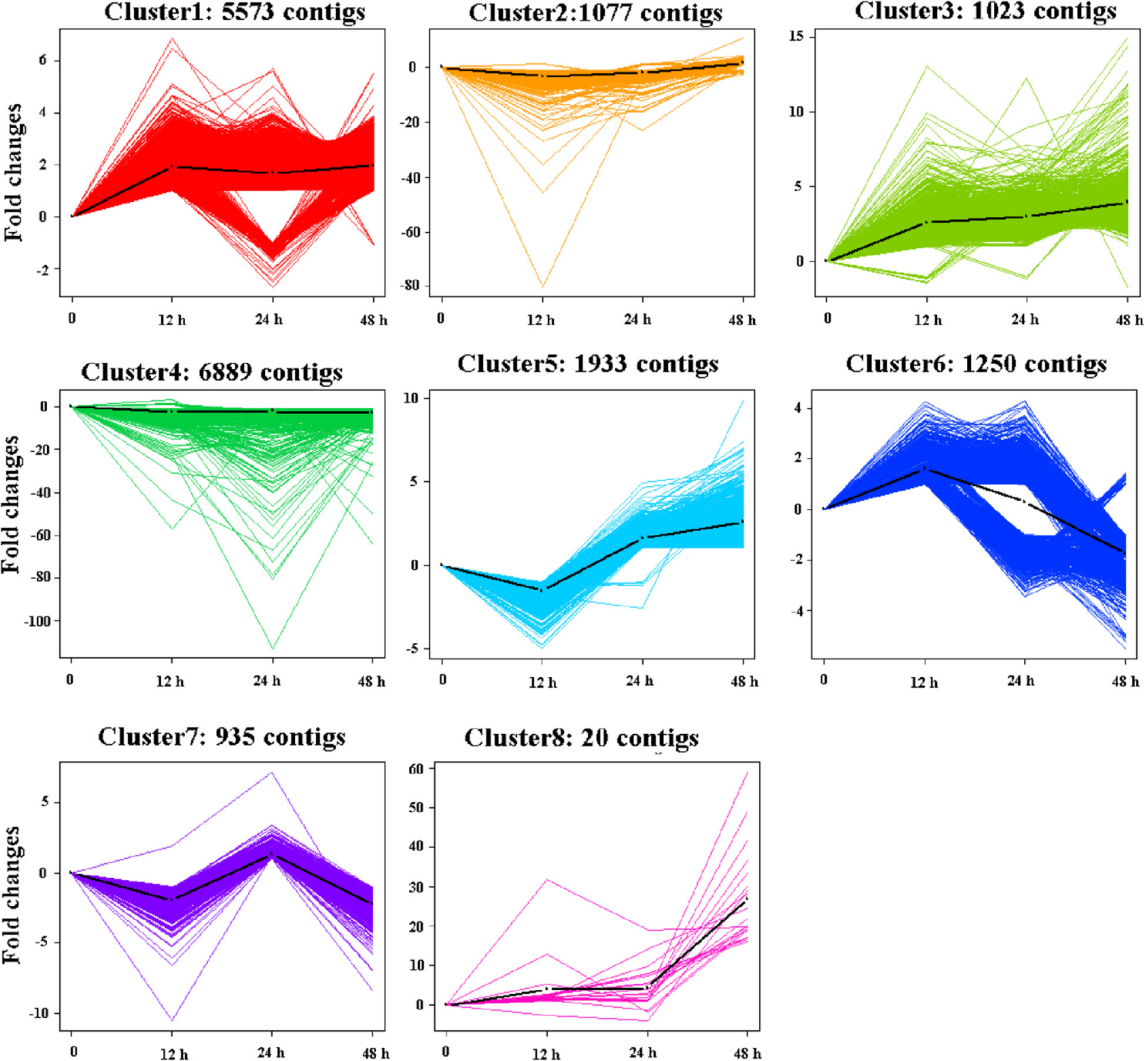
Expression pattern of each cluster of DEGs. A total of 8 clusters were produced; the genes that displayed similar expression pattern were gathered into one cluster

Based on *K*-mean clustering analysis, all contigs were divided into 8 clusters (Fig. 2; Additional file 5). Of these, clusters 1, 3, 5, and 8, which included 5573, 1023, 1933, and 20 contigs, respectively, showed up-regulated patterns of expression within 48 h of algicide exposure. Of these four clusters, cluster 1 was the least up-regulated, with a mean change in expression of around 2-fold and no obvious difference among tested samples. By contrast, expression of contigs in clusters 3 and 8 gradually increased over 48 h, and cluster 8 had greatest increase in expression, which occurred at 48 h. The contigs in cluster 5 were down-regulated at first and then expression gradually increased. Cluster 2 (1077 contigs), cluster 4 (6889 contigs), and cluster 7 (935 contigs) showed no obvious changes or down-regulation. In cluster 6 (1250 contigs), contigs generally displayed up-regulation first and then down-regulation patterns; some contigs showed the highest expression level at 12 h or 24 h (>2 fold changes), and then they showed decreased expression levels i.e., lesser than that of control. Other contigs showed increased expression levels i.e., more than that of control, and then decreased expression levels > 2 fold changes at 48 h.

The up- and down-regulated contigs with > 2 fold changes were analyzed separately. Base on this criterion, we detected 848 up-regulated and 726 down-regulated contigs at all time intervals i.e., 12 h, 24 h, and 48 h (Additional file 6). These data were then combined with *K*-mean clustered contigs with 2-fold changes in expression and overlapping contigs were removed before further analysis. The three up-regulated clusters (clusters 3, 5, and 8) and > 2-fold up-regulated contigs were pooled together into group 1 (3624 contigs in total); and cluster 6 and > 2-fold down-regulated contigs were pooled together into group 2 (1976 contigs in total).

### Classification of DEGs by KEGG analysis

**Fig. 3:**
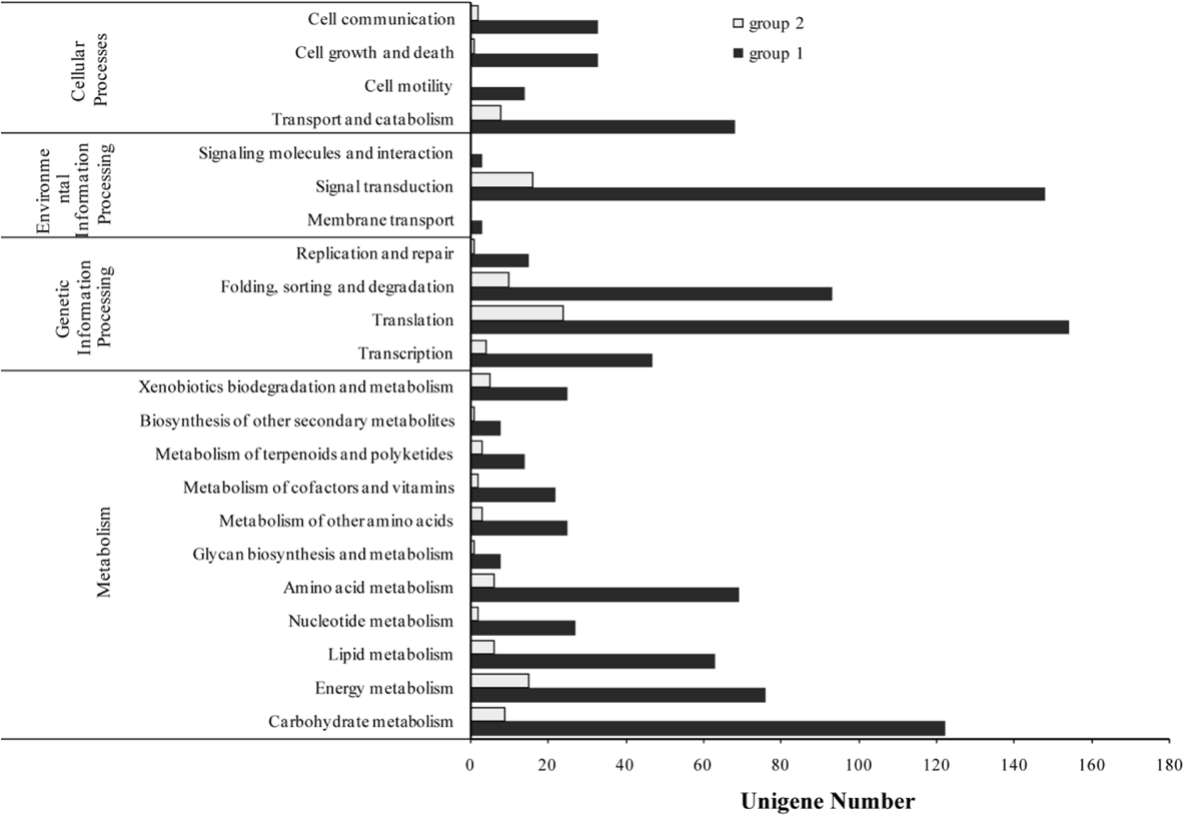
The pathway assignment of DEGs by KEGG pathway analysis. Each group of DEGs was assigned to KEGG database, and gene numbers were got from KEGG online analysis

The contigs that matched to bacteria and viruses were filtered out, and only eukaryote-matched contigs were subjected to further KEGG pathway analyses. This showed that 1130 eukaryote-matched contigs were assigned to group 1, and these contigs mapped to 157 KEGG pathways (Additional file 3) excluding organismal systems and human disease pathways (Fig. 3). These were assigned to metabolic processing (457 genes), genetic information processing (309 genes), environmental information processing (154 genes), and cellular processes (148 genes). The top three second level pathways were translation (154 genes) (Additional file 7), signal transduction (148 genes) (Additional file 8), and carbohydrate metabolism (122 genes). The top three third level pathways were ribosome (76 genes) (Additional file 7), spliceosome (41 genes) (Additional file 9), and oxidative phosphorylation (36 genes) (Additional file 10). Of interest was that in the transcription pathway analysis 47 genes were identified as spliceosome, but only 2 transcription factors were identified.

In group 2, we detected 374 eukaryote-matched contigs; of these, 133 were assigned to the KEGG database, and mapped to 77 KEGG pathways (Fig. 3, Additional file 3) excluding organismal systems and human disease pathways These were assigned to metabolic processing (64 genes), genetic information processing (45 genes), environmental information processing (23 genes), and cellular processes (20 genes). The top three second level pathways were translation (26 genes) (Additional file 7), signal transduction (23 genes) (Additional file 8), and energy metabolism (16 genes). The top three third level pathways were ribosome (20 genes) (Additional file 7), photosynthesis (Additional file 11) (7 genes), and protein processing in endoplasmic reticulum (6 genes). In the transcription pathway, only one gene was assigned to the spliceosome pathway and no transcription factor was detected.

### Photosynthetic and mitochondrial gene responses in *C. polykrikoides*

Using KEGG pathway analyses, we found that the most affected metabolic pathways were oxidative phosphorylation in group 1 (Additional file 10), and photosynthesis in group 2 (Additional file 11). Since not all the sequences were annotated in the KEGG database, we further characterized the genes, which were involved in photosynthetic light reaction and mitochondria, using NR and GO database annotation. In these analyses, contigs that coded for the same gene were counted as single genes.

**Fig. 4:**
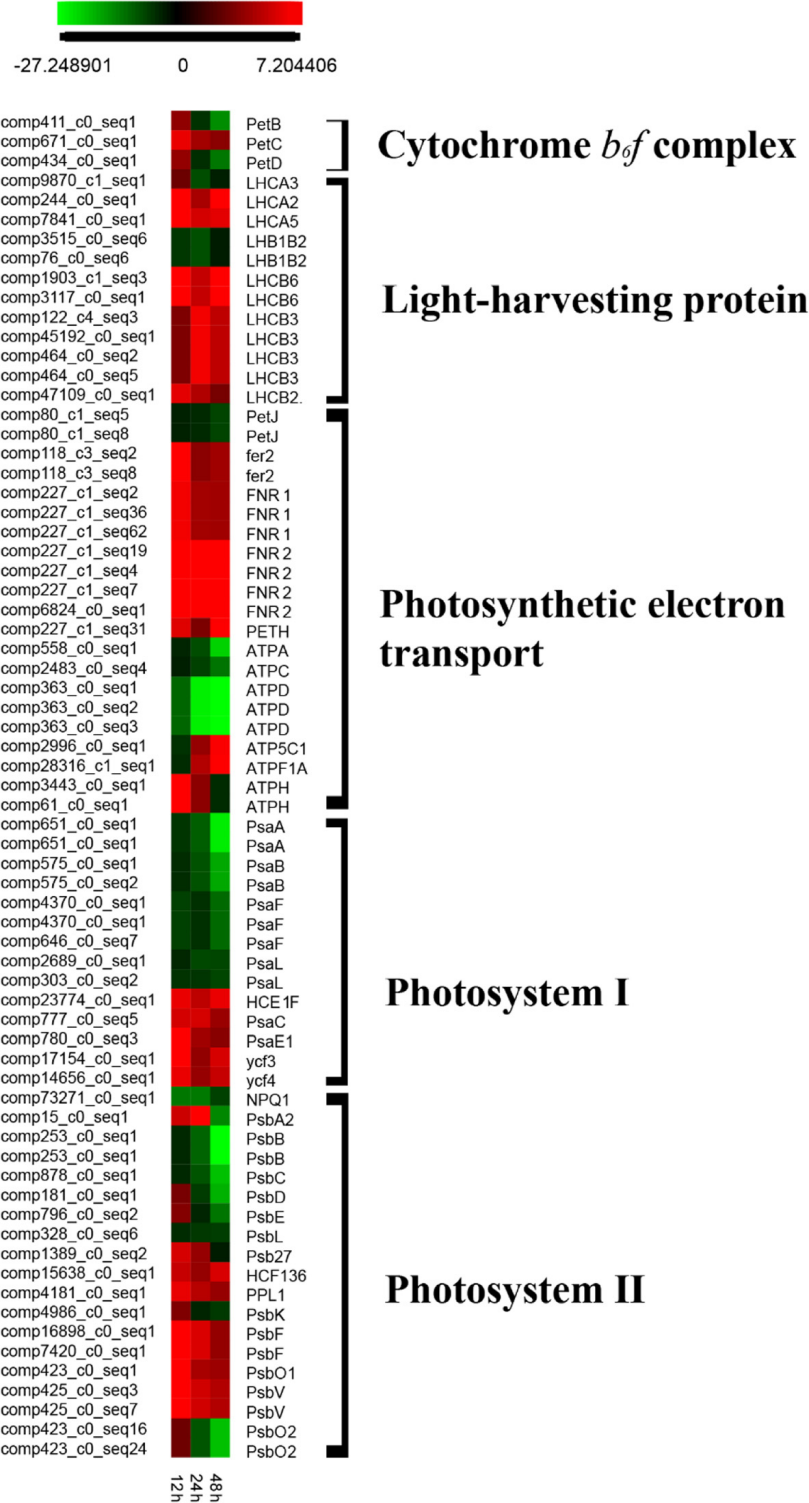
Heat map of DEGs that involved in the photosynthesis light reaction process. Log ratio fold changes were used as relative gene expression level compared to control. The full name of the gene and their characteristic were listed in the Additional file 12

Among regulated photosynthesis genes (Additional file 12; Fig. 4), most genes involved in photosystem I (PS I), photosystem II (PS II), and cytochrome *b*_*6*_*f* complex showed patterns of down-regulation, particularly at 48 h after exposure to copper sulfate. Of a total of nine genes involved in the PS I complex, four genes were down-regulated from 12 h to 48 h after algicide exposure. Other genes were up-regulated and showed highest expression levels at 12 h, and then the expression levels were decreased. Transcriptional expression of the fifteen genes involved in the PS II complex gradually decreased from 12 h or increased at first, and then decreased; nine genes were down-regulated >2-fold at 48 h. As for the chloroplast cytochrome *b*_6_*f* complex, two genes were down-regulated at 48 h, and one gene was up-regulated by >2-fold at 12 h, and expression then decreased. Differing from PS II, PS I, and cytochrome *b*_6_*f* complex, expression of most light-harvesting proteins was up-regulated. Of seven light-harvesting protein genes, three genes were down-regulated > 2-fold at least once (at one time point), and four genes were up-regulated > 2-fold changes at least once. Of eleven photosynthetic electron transport associated genes, expression of four gradually decreased within 48 h; expression of three increased at first and then decreased; two were up-regulated; and two were down-regulated and then increased expression, showing >2-fold changes at 48 h.

**Fig. 5:**
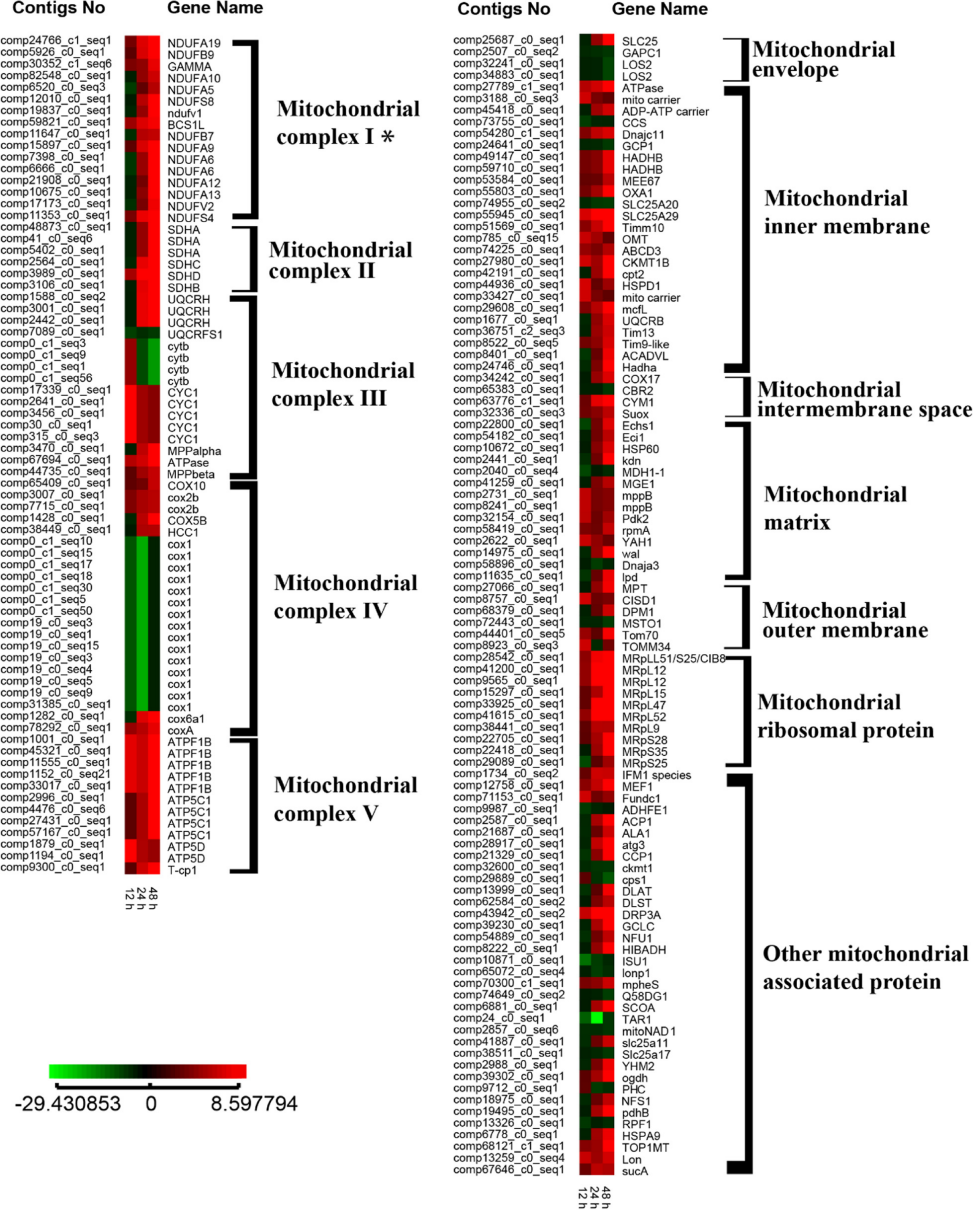
Heat map of DEGs that related to the mitochondria. Log ratio fold changes were used as relative gene expression level compared to control. The full name of the gene and their characteristic were listed in the Additional file 13. “*” indicated the controversial genes that present or lost in the dinoflagellate *C. polykrikoides*

Oxidative phosphorylation is a metabolic pathway that takes place in mitochondria. We examined behavior of certain mitochondrial associated genes which may contribute to mitochondrial composition or have mitochondrion-related functions (Additional file 13; Fig. 5). Of a total of 132 of these genes, 107 were up-regulated by >2-fold. Focusing on mitochondrial complex (MC) I, II, III, IV, and V composition genes, some of MC I and II genes were slightly down-regulated; however, all MC I and II coding genes were up-regulated from 24 h after algicide exposure, attaining >2-fold changes at 48 h. Expression of most of genes that coding for MC III component was increased, and only two genes showed down-regulation patterns. Overall, genes that code for MC IV and V showed up-regulation patterns. These results indicate that the mitochondrial genes examined participate in the cellular response to CuSO_4_ exposure.

### Antioxidant gene responses and copper function genes in *C. polykrikoides*

**Fig. 6:**
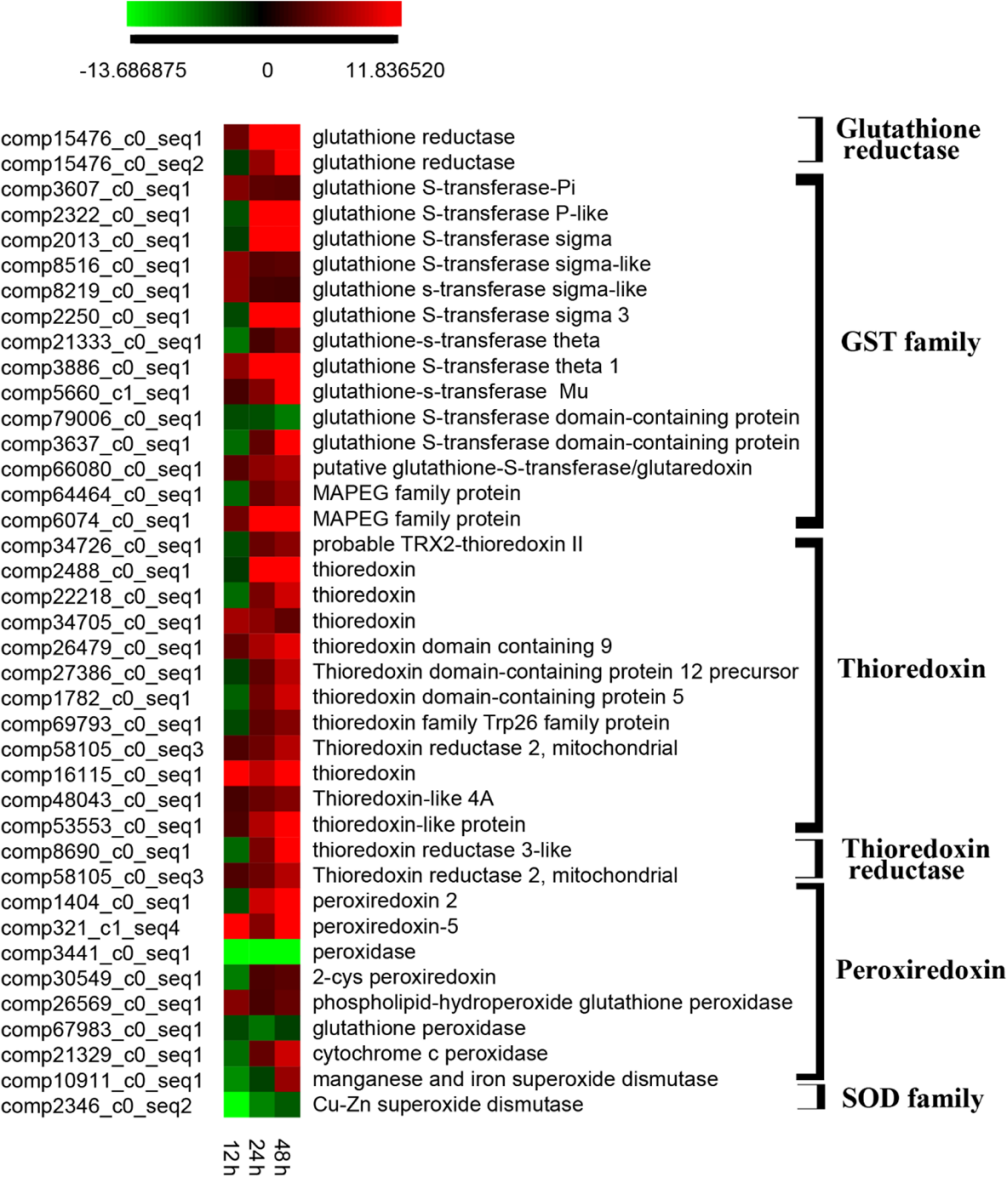
Heat map of DEGs that are coded antioxidant enzymes. Log ratio fold changes were used as relative gene expression level compared to control. The full name of the gene was listed in the Additional file 14

Since CuSO_4_ is a potential reactive oxygen species (ROS) inducer, production of certain antioxidants involved in detoxifying ROS in cells may be affected following exposure. Hence, we examined the behavior of antioxidant genes, including those associated with peroxiredoxin (*Prxs*), glutathione S-transferase (*GST*), glutathione reductase (*GR*), thioredoxin (*Trx*), etc. (Additional file 14; Fig. 6). Of these, expression of *Prxs*, *GST*, *GR* and *Trx* associated genes gradually increased following CuSO_4_ exposure (Fig. 6). In addition, some copper transporting and binding associated genes such as P-type ATPases were found to be expressed differentially (Additional file 15). Furthermore, widely studied chloroplast copper binding protein genes such as Cu/Zn superoxide dismutase (SOD) showed down-regulation patterns at 12 h, and then expression increased a little. Mitochondrial copper binding protein related genes, such as cytochrome C oxidase subunit 17 (*COX17*), *Sco1p* as well as some others were up-regulated following copper sulphate exposure (Additional file 13).

### qRT-PCR validation of DEGs identified by transcriptome sequencing

**Fig. 7:**
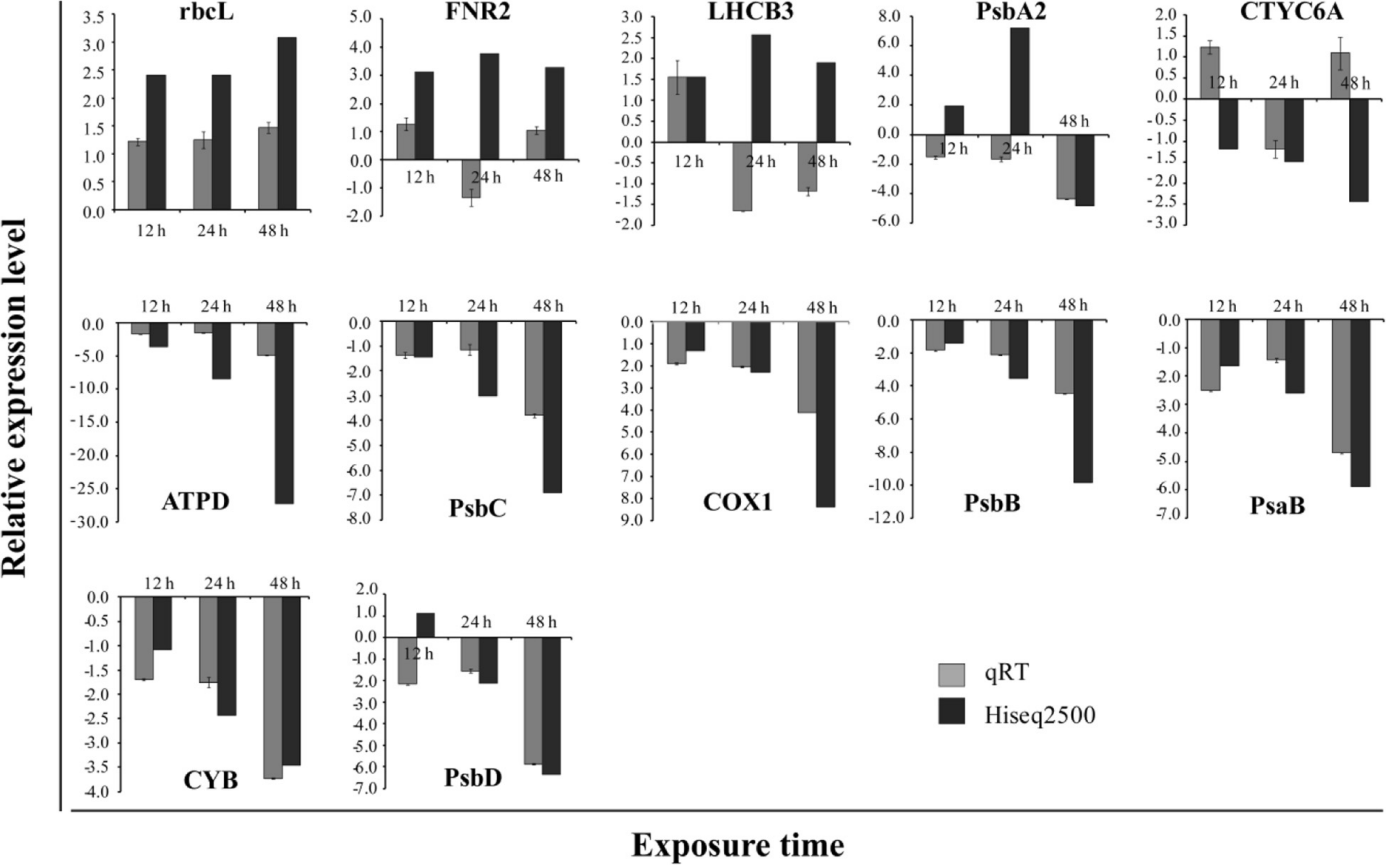
qRT-PCR validation of 13 DEGs identified by RNA-Seq. The relative expression levels of 12 h, 24 h, and 48 h CuSO_4_ treated sample were presented. The housekeeping gene α-tubulin (*TUA*) was used as an internal control for qRT-PCR normalization. The relative expression level of control was considered as 1, and the control samples were not shown in the figure

We selected 13 DEGs with >2 fold changes in expression (Additional file 1) and validated our results using qRT-PCR (Fig. 7). Seven of these genes (*ATPD -* ATP synthase beta, *PsbC-* PSII CP43 apoprotein, *PsaB -* PSI P700 apoprotein, *rbcL-* ribulose 1,5-bisphosphate carboxylase oxygenase form II, *PsbB-* PSII CP47 apoprotein, *COX1 -* cytochrome oxidase subunit 1, and *CYB -* cytochrome b) showed similar results to those obtained via RNA-Seq over the whole test period, with the same expression patterns, but relatively different expression levels. Of the other genes (*FNR2 -* chloroplast ferredoxin-NADP(+) reductase 2, *LHCB3-* light-harvesting complex II *a/b* binding protein 3, *CTYC6A -* chloroplast cytochrome c6, *PsbA2-* PSII D1 reaction center protein, *PetB -* cytochrome b6, and *PsbD -* photosystem II protein D2), one or two tested samples (different time point) of each gene showed different expression patterns. Overall the results showed similar patterns of expression but with different strengths; the Spearman correlation coefficient (*R*) of the tested samples was 0.69 (*N* = 39, *P* < 0.001).

### The effect of CuSO_4_ on *C. polykrikoides* photosynthesis and lipid peroxidation

**Fig. 8:**
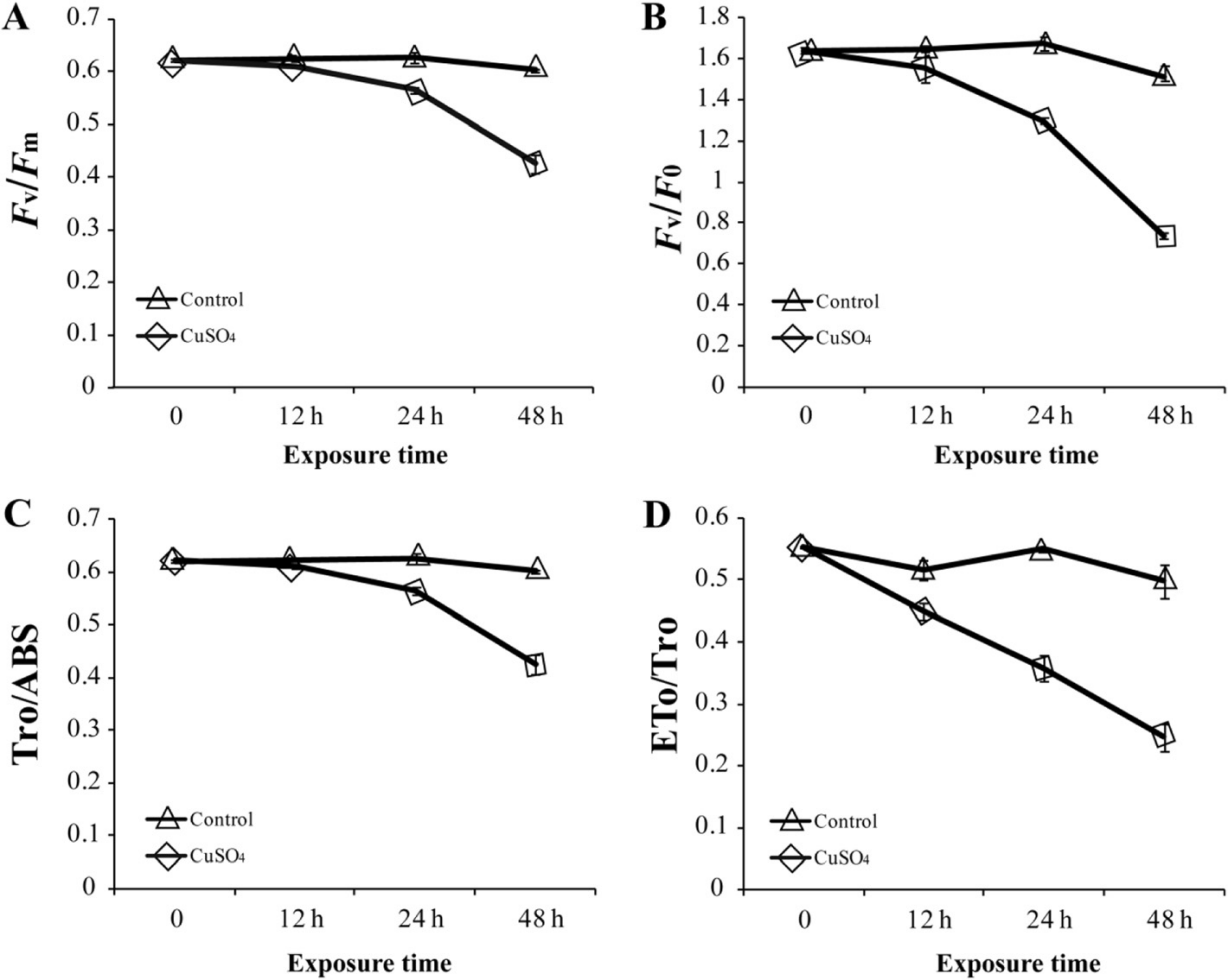
Photosynthesis parameters of *C. polykrikoides*. **a** *F*v/*F*m, maximum quantum efficiency of photosystem II; **b** *F*v/*F*o, a value that is proportional to the activity of the water-splitting complex on the donor side of the PS II; **c** TRo/ABS (ΨEo), represents the maximum yield of primary photochemistry; **d** Eto/Tro (Ψo) represents the efficiency with which a trapped exciton can move an electron into the electron transport chain beyond Q_A_-

Chlorophyll fluorescence parameters were monitored using a pulse amplitude modulation chlorophyll fluorometer. The maximal quantum efficiency of PS II *F*v*/F*m in *C. polykrikoides* decreased considerably with increasing CuSO_4_ exposure time. In addition, *F*v/*F*o, ΨEo, and Ψo gradually decreased with increasing exposure time to CuSO_4_ (Fig. 8).

The lipid peroxidation was increased in *C. polykrikoides* after 6 h and 72 h 1 mg L^−1^ CuSO_4_ exposure (Additional file 16).

## Discussion

HABs caused by the dinoflagellate *Cochlodinium polykrikoides* are a global concern due to their geographic expansion and harmful environmental impacts. However, molecular understanding of the species has received little attention compared to studies of the species focusing on environmental monitoring, physiology, and toxicology. Those studies of HAB-forming species addressing genetic aspects have focused on discovering genes responsible for toxin production and the effects of nutrient availability on the regulation [21, 24, 37, 38]. In this study, we characterized for the first time genome-scale transcriptomes of *C. polykrikoides*, and further analyzed DEGs in response to algicide (copper sulfate) exposure to gain insights into the molecular mechanisms underlying the bloom termination process.

The effect of CuSO_4_ on the *C. polykrikoides* had been tested in several studies [10, 28]. Although the same strain was employed in these studies, the sensitivity of the cell to CuSO_4_ was quite varied. Indeed, *C. polykrikoides* (CP-01) was very tolerant to CuSO_4_, and other contaminants as well in previous generations [28], the tolerance was decreased in more recent generations [10]. The reason might due to differential composition of multi-clone that happened after several successive transfers. Due to clonal variation, the reduction of *Daphnia magna* quite varied after stressor exposure like cadmium or cyanobacteria *Microcystis aeruginosa* [39]. In this study, the CuSO_4_ concentration we have selected was 10 times lower than that of 72 h-EC_50_ [28]. Furthermore, the copper concentration, we selected by considering the World Health Organization’s guideline for copper in drinking water, which was 2.0 mg/L [40].

With the functional annotations presently available in three databases used (NR, GO, KEGG), few contigs could be annotated. The highest number of annotations (82,749 contigs, 43.3 %) was obtained from the NR database. One of reason for this was that some contigs were generated by non-coding 5’- or 3’- untranslated regions, another reason might be the presence of many ‘no-hit’ contigs, belonging to undiscovered novel genes and/or non-coding RNAs (e.g., miRNA, siRNA, and rRNA), that are known to be present in dinoflagellates [41, 42]. However, these annotation success rates were higher than those from available transcriptome data for other dinoflagellates, including *Alexandrium catenella* and *Symbiodinium minutum* [16, 23]. This suggests that the enough of the functional genes in *C. polykrikoides* might have been identified in our experiments to sufficiently characterize the whole genome response of the species.

The KEGG pathway analysis provided physiological pathway information for *C. polykrikoides*. The KEGG pathways of *C. polykrikoides* were quite different from those of other dinoflagellates. For example, in *A. catenella* spliceosome, translation factor, and RNA transport were the top pathways [23]. However, in both of *C. polykrikoides* and *A. catenella*, many genes that were assigned to genetic metabolism or genetic information processes were identified. In addition, both species have many genes assigned to spliceosome, indicating that spliceosome is likely to be crucial in dinoflagellate genetic processes such as RNA-splicing. These transcriptome data provided basic genetic information on *C. polykrikoides*, however, further investigations on the characteristics and functions of *C. polykrikoides* genes are essential.

The mechanism of spliced *trans*-splicing of mRNA and no typical recognized promoter in the dinoflagellates implied that the dinoflagellate genes expression were regulated by post-transcriptional regulation [15, 43, 44]. Furthermore, investigations on some genes and their protein expressions revealed that they were regulated at protein level. These data were consistent with the post-trancriptional regulation hypothesis [17, 45, 46]. In addition, the low amount of transcriptome were identified in response to stress conditions in some dinoflagellates, for example, *Pyrocystis lunula* microarray studies showed that around 5.8 % of DEGs (204 in total of 3500 genes) responded to nitrite, and 1.1 % genes to the herbicide paraquat [18]. These data showed that dinoflagellates have no expression preference for transcriptional gene regulation pattern. However, the little higher expression amount transcriptome were also found in some dinoflagellates. For example, transcriptome analyses of *Alexandrium fundyense* showed that 10 % of signature genes were differentially expressed at two different nutrient conditions [19]. More recently, Johnson et al. [22] reported 29 % of genes were differentially expressed among different life stages in the dinoflagellate *Karenia brevis*. We detected that expression of 20.6 % of contigs changed following exposure to the algicide CuSO_4_. In this context, the percentage of response to CuSO_4_ in this study was not low; this percentage was similar or higher than that shown in other dinoflagellates, suggesting that the algicide might considerably affect molecular genomic processes in *C. polykrikoides*. These HiSeq results were validated in a separate qRT-PCR assay, with significant correlation (*R*^2^ = 0.69, *P* < 0.05) between results from the two methods.

Environmental stress can cause rapid changes in the production of cellular proteins for survival, and responses are controlled at multiple levels, including transcriptional, post-transcriptional, and translational levels [47]. As a protein involved in genetic processes, ribosome is central to the translation process; ribosomal proteins have functions not only in ribosome composition, but also involved in various regulation processes. Hence, their expression is regulated to balance the protein production in response to environmental changes [48, 49]. In this study, KEGG pathway analyses showed that many ribosomal protein genes were regulated by CuSO_4_. Similar results have been found in other various organisms, such as fungi, plants, and algae [48, 49]. For example, the ribosomal protein L44 gene was up-regulated by salt, sorbitol, and heavy metal exposure in the fungus *Aspergillus glaucus* [49]. Differential expression of cytosolic ribosomal protein genes was induced by CuCl_2_ in marine algae *Ulva compressa* [50], by various environmental conditions (elevated sugar, nitrogen stress, and UV exposure) in *Arabidopsis thaliana* [48], and even at different life stages of the dinoflagellate *K. brevis* [22]. In this study, we found that some differentially expressed ribosomal protein genes, such as those coding for ribosomal proteins L44 (*RPL44*) and *RPL11*, were up-regulated by exposure to CuSO_4_. Taken together with previous findings, our results suggest that ribosomal protein genes (e.g., *RPL44*, *RPL11*) may be involved in cellular regulation in response to algicide exposure in dinoflagellates.

Transcription factors commonly regulate gene transcription in various cellular processes [51, 52]. In this study, only a few transcription factors were detected by DEG analysis. This implies that transcription factors were not widely involved in the gene regulation of *C. polykrikoides* when exposed to CuSO_4_. This provides further evidence of unusual gene regulation patterns in dinoflagellates, and highlights the need for further investigation of the mechanisms of gene regulation in this group. Nevertheless, these findings suggest that the algicide CuSO_4_ may disrupt gene transcription and translation in *C. polykrikoides*, slowing cell growth, accelerating cell death, and thereby inducing HAB termination.

Genes involved in photosynthesis in plants and algae are sensitive to various environmental stimuli, such as excess light, salinity, metals, etc. [53–55]. The transcription of photosystem genes was decreased in the diatom *Ceratoneis closterium* when exposed to copper [53] and in the green algae *Chlorella* when treated with Pb^2+^ [55]. In our DEG analyses, core photosystem coding proteins (e.g., *PsaA* and *PsaB* of PS I, *PsbB* of PS II) were also down-regulated at 12 h after algicide exposure. In contrast, expression of some other photosynthesis-related genes, such as those coding for light harvesting proteins (e.g. *LHB3*, *LHB6*), along with *PsaC* (PS I), and *PsbF* (PS II) was increased. Similar results have been found in salt stressed rice, the opposite alteration of genes expression pattern of *PsaD* genes and other tested genes including *PsaH*, *LHCA1*, *LHCA2* and *LHCA4* were found in the salt stressed rice [56]. In addition, in herbicide (atrazine, bentazon) treated soybean expression of PS II type I and type II chlorophyll *a*/*b* binding proteins was decreased, while *PsbR* and *PsbS* genes, and the oxygen evolving complex were up-regulated [57]. In dichloromethane and dichloroethane treated cells of the algae *Chlorella*, *PsaB* genes were up-regulated in the first 12 h, but were down-regulated after 24 h [58]. These results suggest that differential expression of photosystem genes under stress, and that in *C. polykrikoides* the photosystem apparatus (e.g., PS I and PS II) may be experience ongoing damage with increasing exposure time and doses of CuSO_4_.

The damage of photosystem by CuSO_4_ was also supported by our results showing the gradually decreasing photosynthetic efficiency (Fig. 8). This is in accordance with the results of previous research, which showed reduction in both *F*v*/F*m and *F*v/*F*o in the black mangle when exposed to copper [59]. Additionally, in the red algae *Antithamnion plumula*, *F*v*/F*m decreased and PS II activity gradually decreased with exposure to increasing concentrations of Cu^2+^ [60]. These findings, taken together with the results of this study, suggest that excess Cu^2+^ might affect photosystem gene regulation and inhibit photosynthetic efficiency in dinoflagellates. These adverse effects might be due the direct damage to PS II or the inhibition of PS II repair mechanisms [61].

In contrast to photosynthesis-associated genes, we found that most mitochondrial genes were up-regulated in *C. polykrikoides* when exposed to CuSO_4_ (see Fig. 5). This is in agreement with the results of a previous study showing that the mitochondrial genes *COX11* and *COX17* were up-regulated in the algae *Ulva compressa* when exposed to copper [50]. This increased expression of mitochondrial genes may take place due to damage to mitochondrial proteins [50], or to boost oxidative phosphorylation to increase the probability of cell survival [62, 63]. In addition, many stress responses, such as rebalancing of cellular metabolite concentrations and altered ROS abundance, can be related to mitochondrial processes [63]. Robust mitochondrial function may be crucial to the survival of *C. polykrikoides* following algicide exposure, and prevention of reduced photosynthetic efficiency depends upon proper mitochondrial function under such stress conditions [63].

Mitochondria and photosystem require copper for their metabolic functions; however, excess copper can induce ROS production and is toxic to most organisms [64, 65]. Indeed, the disturbance of mitochondria and photosynthesis-related genes is an indication of induced ROS production, since these are the organelles that produce intracellular ROS [62, 66]. In this study, we measured ROS production in *C. polykrikoides* exposed to copper using fluorescence spectrometry. However, we could not quantify the exact amounts of ROS produced, because the naked CuSO_4_ treated dinoflagellate cells were highly fragile and were easily destroyed during ROS sample preparation. This considerable ROS production was in agreement with the results obtained for another dinoflagellate, *Prorocentrum minimum*, exposed to the same algicide, CuSO_4_ [65, 67]. It is likely that that copper induces oxidative stress by facilitating the generation of ROS, and high doses of copper damage *C. polykrikoides* cells irreversibly [10, 65].

Such cellular oxidative stress can be mitigated by the activation of antioxidant systems [68]. The main antioxidants include glutathione (GSH), thioredoxin (Trxs), peroxiredoxin (Prx), superoxide dismutase (SOD), and catalase [10, 69–71]. In this study, most up-regulated antioxidant genes were related to GSH, Trxs, and Prx production. The up-regulation of antioxidant enzyme (e.g. Prx, Trxs, and GSH) production was increased following copper exposure in the marine algae *Ulva compressa* [50] and also in the dinoflagellate *P. minimum* [65]. In particular, Glutathione S-transferase (*GST*) is a large gene superfamily, the members of which function as antioxidants by conjugating GSH to toxic substances. In addition, differential transcriptional expression of *GST* members in response to variation in environmental conditions has been found in various organisms [65, 72, 73]. Increased expression of MAPEG (membrane-associated proteins in eicosanoid and glutathione metabolism) family members has been reported in *P. minimum* in response to exposure to CuSO_4_ [65]. In addition, reduced GSH production was increased in *P. minimum* cells treated with CuSO_4_ [65]. These results suggest that in dinoflagellates *GST* genes may play an important role in cellular responses to CuSO_4_ induced stress.

The thioredoxin system includes two antioxidants, Trxs and thioredoxin reductase (TrxR) [74]. Trxs is a protein with a conserved redox site that maintains the intracellular redox state and reduces protein thiols. TrxR is required for the reduction of the active disulfide site in Trx, and plays a role in redox regulation [69]. Prxs family genes also function by via a thiol redox mechanism; they respond to various environmental stresses, such as H_2_O_2_, high light, and nutrient deprivation, and they are sensitive to cellular oxidation [75]. Hence, the accumulation of oxidized Prxs may indicate disruption of cellular redox homeostasis [75, 76]. The regulation of *Trx* and *TrxR*, as well as *Prxs* genes may be involved in the response of *C. polykrikoides* to CuSO_4_. Similarly, the copper induced expressions of thioredoxin and Prxs genes or their proteins were found in other organisms. For example, the expression of thioredoxin increased by copper in brown algae *Ectocarpus siliculosus* [77]; expression level of Prx protein increased by copper in macroalgae *Scytosiphon gracilis* [78]; the Prx enzyme activity was increased in brown alga *Dictyota kunthii* by excess copper [79]. Furthermore, Prx likely acted as an active antioxidant to control lipid peroxidation generated by copper in the *S. gracilis* and *D. kunthii* [78, 79]. Corresponding to increased Prxs gene expression, the lipid peroxidation increased in *C. polykrikoides* after CuSO_4_ exposure, which suggests that the Prxs might function in lipid peroxidation by decreasing CuSO_4_ induced stress in *C. polykrikoides*. Because glutathione, thioredoxin, Prxs are all elements of the thiol-disulfide redox regulatory network. This suggests that the thiol-disulfide redox system might play a vital role in the CuSO_4_ induced stress defense network in dinoflagellates.

## Conclusions

This study is the first to report transcriptome profiles of the dinoflagellate *C. polykrikoides* with a focus on the genome-wide molecular responses to the biocide CuSO_4_. CuSO_4_ significantly decreased photosynthetic efficiency and induced widespread regulation of gene networks and signal transduction pathways in *C. polykrikoides* cells. DEG analyses showed that various photosynthetic genes were regulated in *C. polykrikoides* exposed to CuSO_4_. Based on these results, we conclude that the photosynthetic machinery might be severely affected when treated with the algicide, leading cell death. In addition, gene translation and transcription processes may be disturbed, and this may further inhibit cell growth and proliferation, possibly accelerating cell death. However, antioxidant systems and mitochondrial genes are likely to be activated in response to the cellular stress caused by CuSO_4_ exposure, and this might compensate for the decrease in photosynthetic efficiency. These results provide an understanding of the molecular basis of the cellular and genomic responses of HAB forming dinoflagellate cells exposed to algicides, and of the HAB termination process.

## Additional files

Additional file 1:
**The primers and full name of genes that used in the qRT-PCR validation.** (XLSX 12 kb) Additional file 2:
**The contigs length distribution of the** ***C. polykrikoides*** **transcriptome.** The gray bar indicated the total contigs, and white indicated contigs that have annotation in NR database. (PPTX 174 kb) Additional file 3:
**KEGG pathways of DEGs.** The third level pathways of group 1 and group 2 were list in the excel file. Gene numbers indicated the total gene numbers that assigned to each third level pathway. (XLSX 16 kb) Additional file 4:
**The numbers of DEGs.** The DEGs numbers with 2.0-fold cut-off at 12 h, 24 h, and 48 h. (PPTX 82 kb) Additional file 5:
***K*** **-means clustering heat map of DEGs.** Total 8 clusters were shown; the cluster 8 was not recognized clearly since there were only 20 contigs. (PPTX 187 kb) Additional file 6:
**Analysis of contigs showing fold change more than 2.0-fold.** (A) Up-regulation; (B) Down-regulation. (PPTX 157 kb) Additional file 7:
**DEGs that involved in translation.** The genes were identified by KEGG pathway analysis. (XLSX 37 kb) Additional file 8:
**DEGs that involved in signal transduction.** The genes were identified by KEGG pathway analysis. (XLSX 34 kb) Additional file 9:
**DEGs that involved in spliceosome.** The genes were identified by KEGG pathway analysis. (XLSX 14 kb) Additional file 10:
**DEGs that involved in oxidative phosphorylation.** The genes were identified by KEGG pathway analysis. “*” indicated the controversial genes that present or lost in the dinoflagellate *C. polykrikoides*. (XLSX 17 kb) Additional file 11:
**DEGs that involved in photosynthsis.** The genes were identified by KEGG pathway analysis. (XLSX 12 kb) Additional file 12:
**Differentially expressed photosynthesis related genes.** The genes were manually summarized by reviewing GO and NR annotation. (XLSX 22 kb) Additional file 13:
**Differentially expressed mitochondria related genes.** The genes were manually summarized by reviewing GO and NR annotation. “*” indicated the controversial genes that present or lost in the dinoflagellate *C. polykrikoides*. (XLSX 34 kb) Additional file 14:
**Differentially expressed antioxidant genes.** The genes were manually summarized by reviewing GO and NR annotation. (XLSX 14 kb) Additional file 15:
**Differentially expressed copper binding or transport genes.** The genes were manual summarized by reviewing GO and NR annotation. (XLSX 14 kb) Additional file 16:
**Lipid peroxidation levels.** Lipid peroxidation levels expressed as malondialdehyde (MDA) *C. polykrikoides* after 6 and 72 h exposure. Significant differences between the control and treated groups, as determined using one-way ANOVA, are highlighted ***P* < 0.01. (PPTX 163 kb)

## Abbreviations

ATPD: ATP synthase beta
COX (11; 17): Cytochrome c oxidase assembly protein subunit (11; 17)
COX1: Cytochrome oxidase subunit 1
CuSO_4_: Copper sulfate
CYB: Cytochrome b
CYTC6A: Chloroplast cytochrome c6
DEGs: Differentially expressed genes
FNR2: Chloroplast ferredoxin-NADP(+) reductase
FPKM: Fragments per kilobase of transcript per million mapped reads
GO: Gene ontology
GSH: Glutathione
GST: Glutathione S-transferase
HABs: Harmful algal blooms
KEGG: Kyoto Encyclopedia of Genes and Genomes
LHCA (1, 2, 4): Light-harvesting complex I chlorophyll a/b binding protein (1, 2, 4)
LHCB (3, 6): Light-harvesting complex II chlorophyll a/b binding protein (3, 6)
MC: Mitochondrial complex
PetB: cytochrome b6
NR: Non-redundant protein
Prx: Peroxiredoxin
PS I: Photosystem I
PS II: Photosystem II
PsaA: PS I P700 chlorophyll a apoprotein A1
PsaB: PSI P700 apoprotein A2
PsaC: PS I subunit VII
PsaD: PS I subunit II
PsaH: PS I subunit VI
PsbA2: PSII D1 reaction center protein
PsbB: PSII CP47 apoprotein
PsbC: PSII CP43 apoprotein
PsbD: photosystem II protein D2
PsbF: PS II cytochrome b559 subunit beta
PsbR: PS II cytochrome b559 subunit beta
PsbS: PS II 22 kDa protein
rbcL: Ribulose 1,5-bisphosphate carboxylase oxygenase form II
SBH: Single-directional best hit
SOD: Superoxide dismutase
TrxR: Thioredoxin reductase
Trxs: Thioredoxin
