# Supplementary figures and images for: Transcriptomic profiles reveal the genome-wide responses of the harmful dinoflagellate Cochlodinium polykrikoides when exposed to the algicide copper sulfate
Source: BMC Genomics. 2016 Jan 5;17:29. doi: 10.1186/s12864-015-2341-3 (PMC4702327; doi:10.1186/s12864-015-2341-3)

## Slide 1
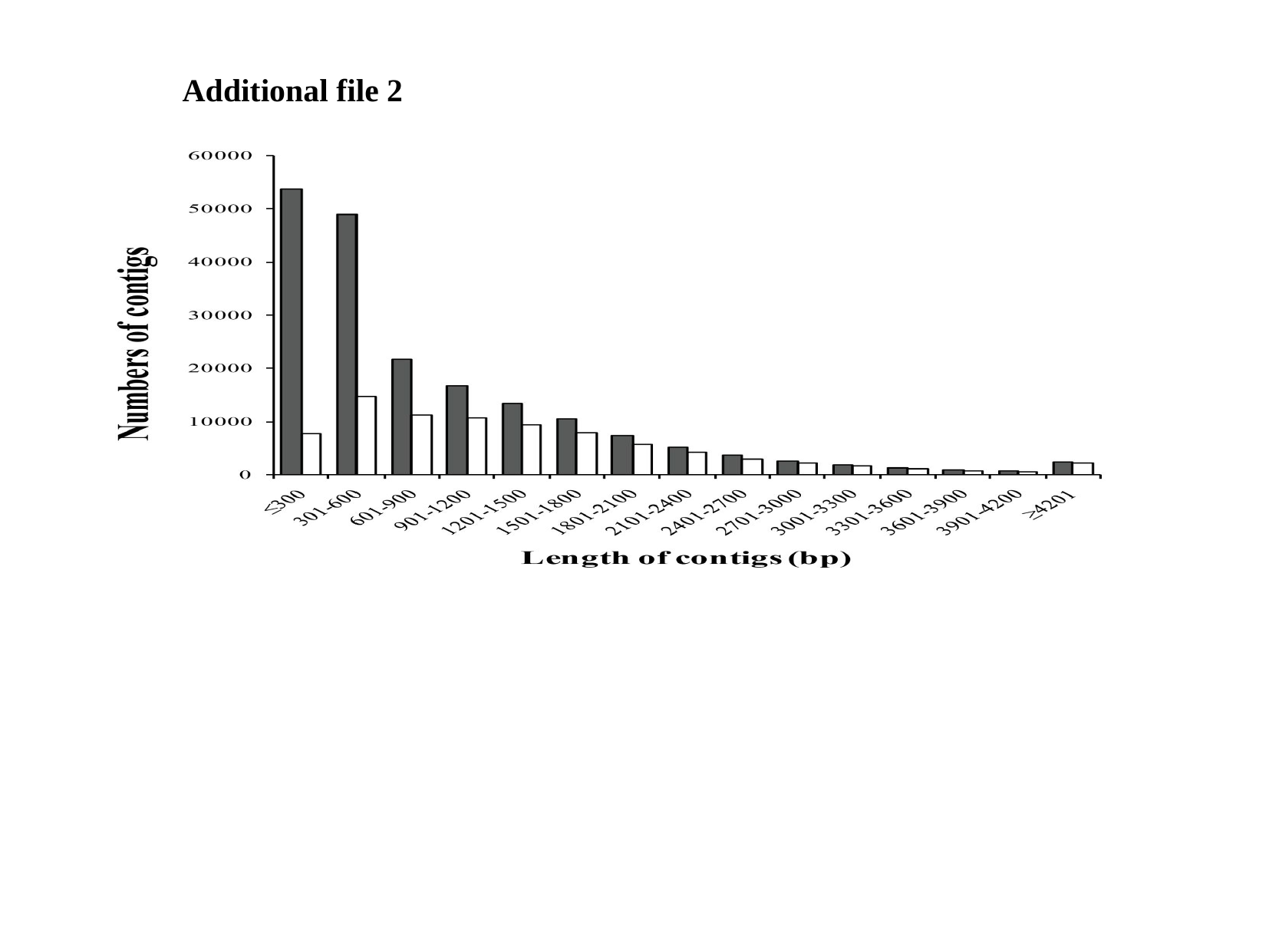

Supplement: Additional file 2: — The contigs length distribution of the C. polykrikoides transcriptome. The gray bar indicated the total contigs, and white indicated contigs that have annotation in NR database. (PPTX 174 kb) [file 12864_2015_2341_MOESM2_ESM.pptx]

## Slide 1
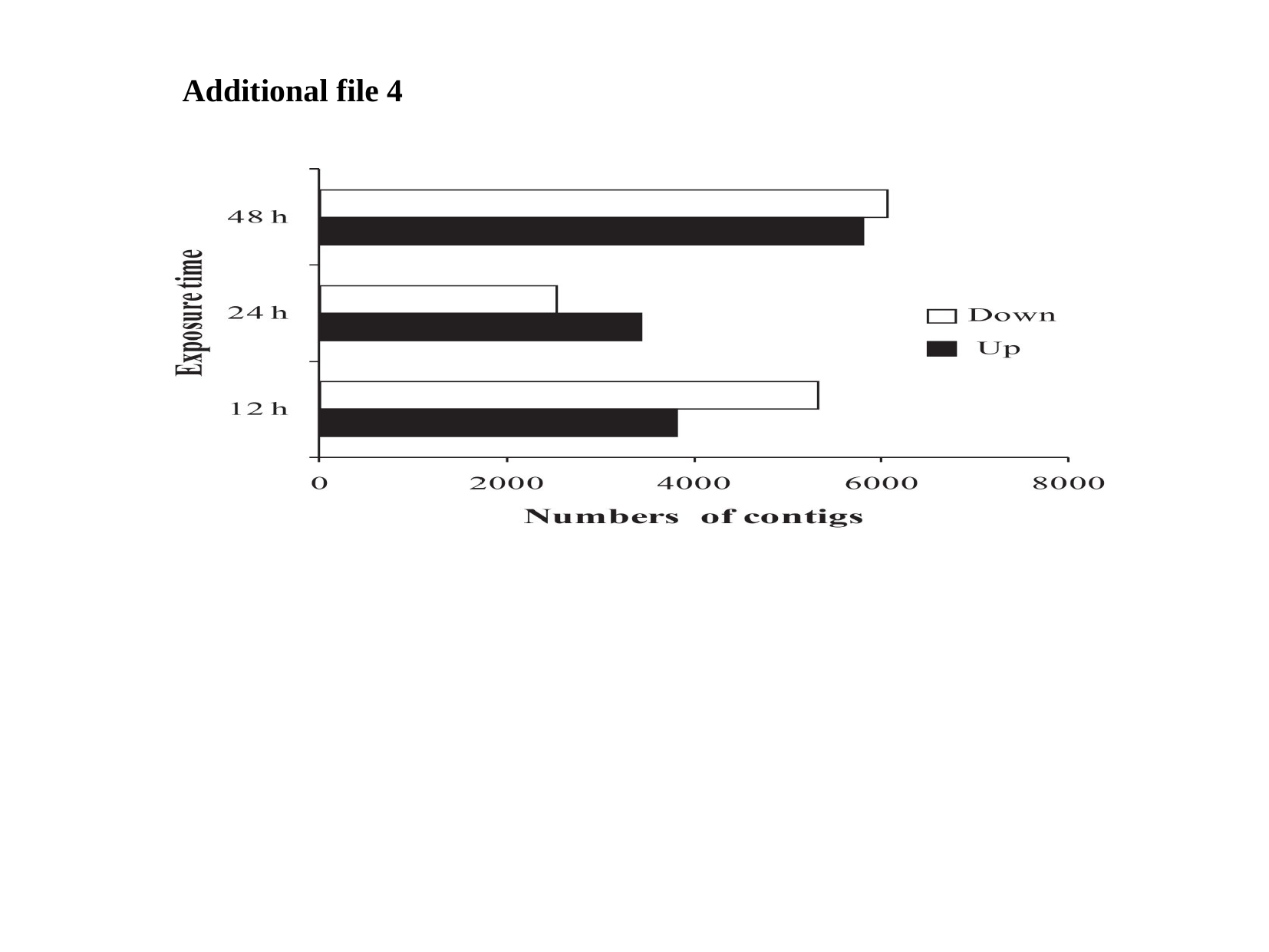

Supplement: Additional file 4: — The numbers of DEGs. The DEGs numbers with 2.0-fold cut-off at 12 h, 24 h, and 48 h. (PPTX 82 kb) [file 12864_2015_2341_MOESM4_ESM.pptx]

## Slide 1
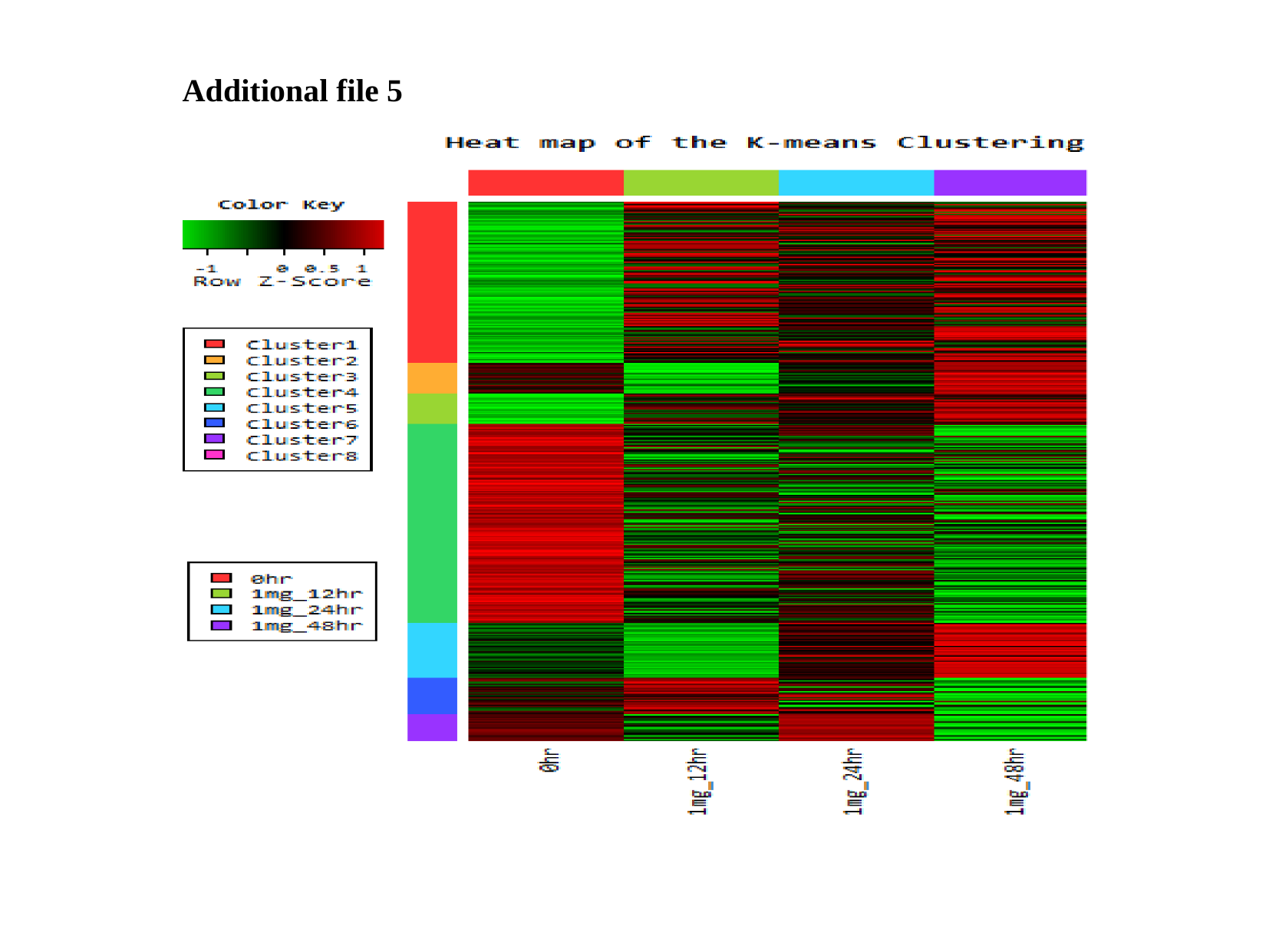

Supplement: Additional file 5: — K -means clustering heat map of DEGs. Total 8 clusters were shown; the cluster 8 was not recognized clearly since there were only 20 contigs. (PPTX 187 kb) [file 12864_2015_2341_MOESM5_ESM.pptx]

## Slide 1
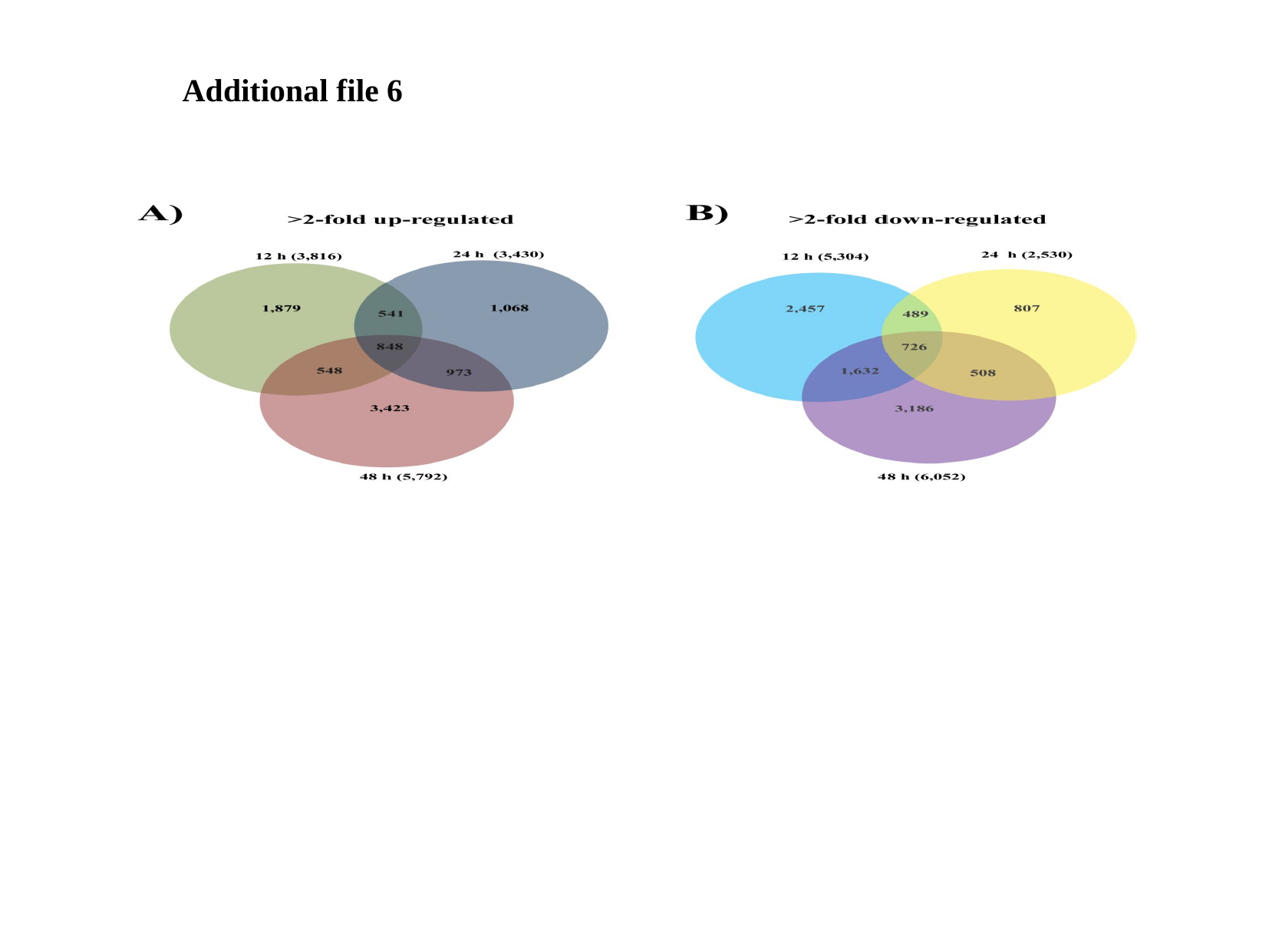

Supplement: Additional file 6: — Analysis of contigs showing fold change more than 2.0-fold. (A) Up-regulation; (B) Down-regulation. (PPTX 157 kb) [file 12864_2015_2341_MOESM6_ESM.pptx]

## Slide 1
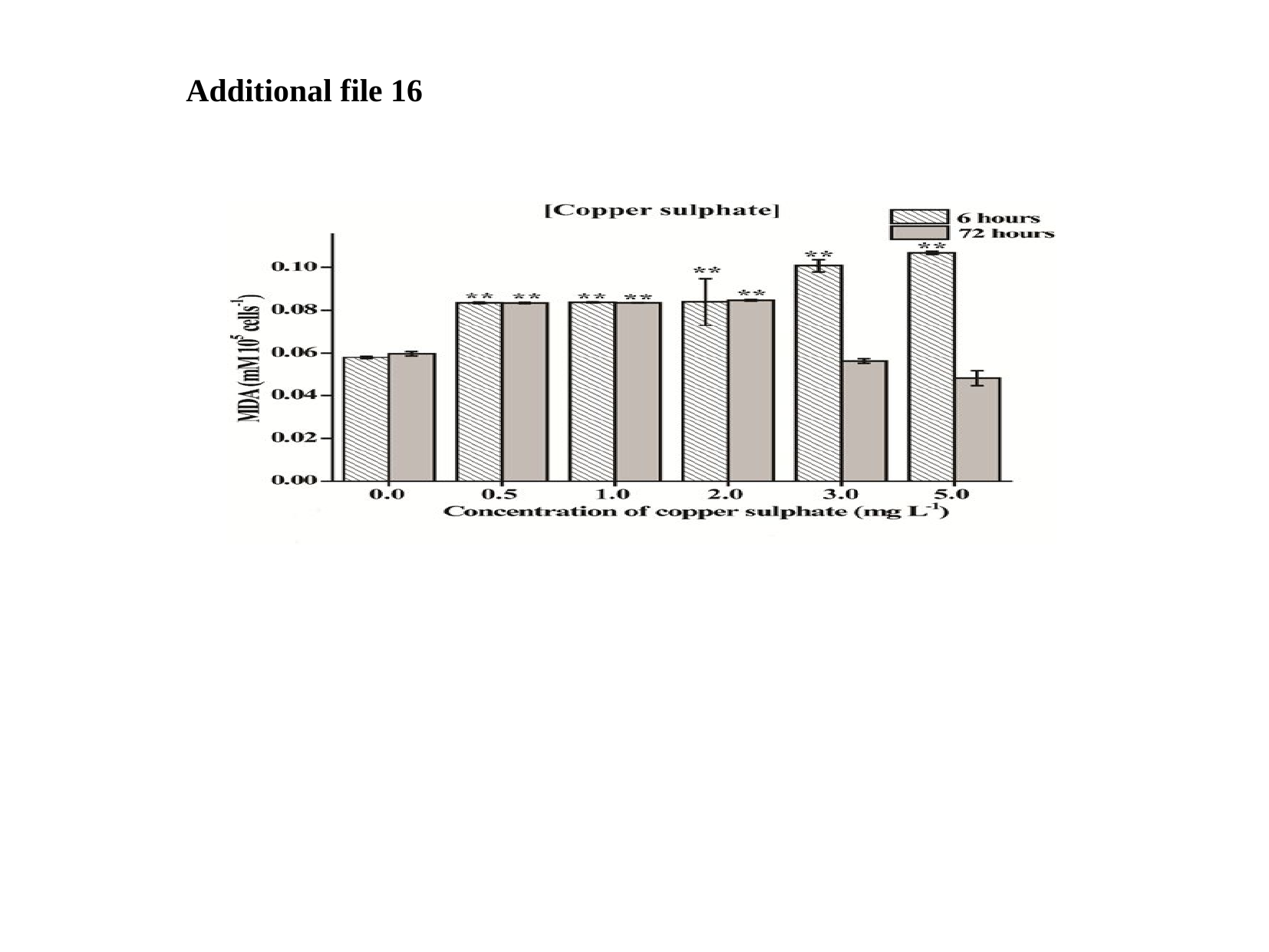

Supplement: Additional file 16: — Lipid peroxidation levels. Lipid peroxidation levels expressed as malondialdehyde (MDA) C. polykrikoides after 6 and 72 h exposure. Significant differences between the control and treated groups, as determined using one-way ANOVA, are highlighted **P < 0.01. (PPTX 163 kb) [file 12864_2015_2341_MOESM16_ESM.pptx]
